# High-Dose Benzylpenicillin Treatment-Induced Febrile Neutropenia in HIV-Infected Male with Neurosyphilis: Case Report

**DOI:** 10.3390/antibiotics14060560

**Published:** 2025-05-30

**Authors:** Inga Sabeckyte-Boveiniene, Kotryna Krupeckaite, Svajune Petkeviciute, Evelina Pukenyte, Aukse Mickiene, Danguole Vaznaisiene

**Affiliations:** 1 Infectious Diseases Department, Lithuanian University of Health Sciences, 47144 Kaunas, Lithuaniaevelina.pukenyte@lsmu.lt (E.P.);; 2 Infectious Diseases Sector, Utena Hospital, 28151 Utena, Lithuania

**Keywords:** neurosyphilis, HIV, neutropenia

## Abstract

**Background**: Prevention of an irreversible sequalae in neurosyphilis patients requires immediate high-dose intravenous benzylpenicillin administered for a prolonged period of time. However, life-threatening neutropenia has been reported as one of the complications following extended usage of benzylpenicillin. **Case presentation**: We report a 54-year-old male patient with HIV who developed high-dose benzylpenicillin-induced febrile neutropenia during neurosyphilis treatment. The patient developed a fever of up to 39.8 °C, severe leukopenia (<1 × 10^9^/L), and neutropenia (0.2 × 10^9^/L). He also presented with slightly elevated C-reactive protein and procalcitonin levels but had no clear symptoms of other infections. The diagnosis was confirmed by excluding other possible causes of neutropenia: flu, measles, sepsis, and HIV-related neutropenia. Third-generation antipseudomonal cephalosporin in combination with vancomycin and granulocyte colony-stimulating factor were administered, and the patient saw a rapid improvement in clinical symptoms and laboratory findings. **Conclusions**: High-dose benzylpenicillin-induced neutropenia should be considered a complication after prolonged periods of neurosyphilis treatment with high-dose benzylpenicillin when there is no evidence of other potential causes of neutropenia. Early diagnosis and proper treatment are critical in order to prevent this dangerous condition from deteriorating further.

## 1. Introduction

Prompt therapy with high-dose intravenous benzylpenicillin for a prolonged period is critical for neurosyphilis patients to avoid irreversible sequelae, such as cognitive impairment, motor dysfunction, and other serious neurological deficits. Early and adequate treatment significantly improves prognosis and reduces the risk of long-term complications. However, life-threatening neutropenia has been reported as a complication of high-dose benzylpenicillin therapy [1,2]. This condition is quite uncommon and occurs in approximately 2.42% of neurosyphilis cases treated with high-dose benzylpenicillin [1,2]. The definite diagnosis could be difficult to establish due to nonspecific symptoms and other possible causes of neutropenia, including infection, neutropenia induced by other medications, nutrition, hematologic malignancies, rheumatologic disorders, autoimmune neutropenia, etc. A thorough evaluation, including patient history, laboratory tests, and, occasionally, a bone marrow biopsy, is important to determine the cause and guide treatment.

## 2. Case Presentation

A 54-year-old man was referred to the hospital with a 3-week history of headache, nausea, and vomiting. He had lost 8 kg in 3 months. The patient did not measure his temperature while at home. He had complained of migraines previously, but he had never suffered migraine episodes of such severity before.

**Figure 1:**
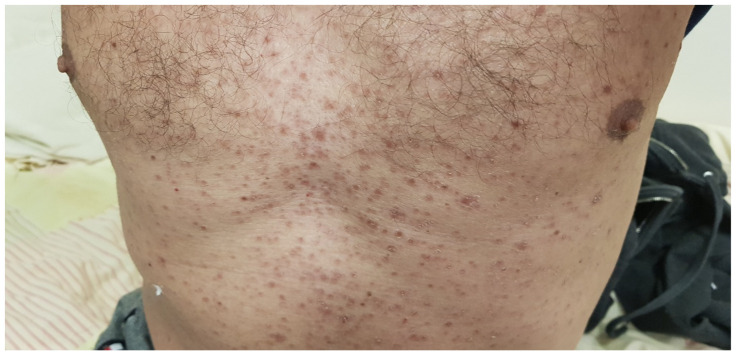
Rash on the chest on admission.

Initially, he was admitted to the emergency department due to disorientation. He could not remember how he got there. A detailed physical examination showed a fever of 37.6 °C, impaired consciousness, with a Glasgow Coma Scale (GCS) score of 12, disorientation, neck rigidity, and no focal neurological symptoms. His hemodynamic response was stable and lung auscultation was normal. A maculopapular rash of an unknown duration was observed on the chest and abdomen area (Figure 1). His eye examination did not reveal any abnormalities.

Ultrasound of the abdominal organs and lymph nodes was performed, detecting enlarged lymph nodes in the neck, armpits, and groin. The chest X-ray was normal.

Laboratory tests showed mild elevation of C-reactive protein (CRP) levels at 24.23 mg/L (reference range: 0–5 mg/L), a normal white blood cell count (WBC) of 4.6 × 10^9^/L (reference range: 4.0–11.0 × 10^9^/L), and a normal platelet count of 158 × 10^9^/L (reference range: 150–400 × 10^9^/L). An urgent computed tomography (CT) scan of the head was performed and demonstrated no definite acute intracranial hemorrhage, midline shift, or mass. A cerebrospinal fluid (CSF) examination revealed 71 × 10^6^ cells/L (lymphocytes 73.2%), total protein 1.01 g/L (reference range: 0.15–0.45 g/L), lactate 5.2 mmol/L (reference range: 1.2–2.1 mmol/L), and glucose 0.59 mmol/L. He was hospitalized in the Department of Infectious Diseases for a more detailed examination and treatment.

The patient’s medical history revealed that, a month ago, he was treated for an unspecified rash on the chest, limbs, and face. A skin biopsy was performed and reported nonspecific dermatitis. The rash improved after corticosteroid, antibiotic, and antihistamines treatment. His previous medical and socioeconomic history was unremarkable, except for the fact that his mother had suffered from tuberculosis.

Ceftriaxone (2 g twice a day) and ampicillin (3 g four times a day) were administered empirically in combination with acyclovir 750 mg (every 8 h).

The next day, the spinal tap was repeated. CSF analysis revealed the following: an 89 × 10^9^ cell count, 1.34 g/L total protein, 1.72 mmol/L glucose, and 2.9 mmol/L lactate. To establish the possible etiology of neuroinfection, the following tests were performed: (1) CSF polymerase chain reaction (PCR) for enteroviruses, herpes simplex 1 and 2, varicella-zoster virus, cytomegalovirus, Epstein–Barr virus, human herpesvirus-6, human herpesvirus-7, human herpesvirus-8 (negative); (2) CSF staining for acid-fast bacteria (negative); (3) CSF cytological examination (did not show atypical cells); and (4) CSF and blood cultures (negative). Serology tests for tick-borne encephalitis (IgM and IgG) were also negative.

Subsequent investigations confirmed the diagnosis of syphilis, as his rapid plasma reagin test was reactive in a 1:128 dilution, and his Treponema pallidum hemagglutination assay test was positive (4+). The CSF Venereal Disease Research Laboratory test was also positive (1:4). The serological test for HIV was positive as well. After HIV serology came back positive, the patient confessed that he was diagnosed with HIV infection eight years ago and has been taking abacavir/lamivudine and efavirenz treatment. His last CD4 cell count was >500 cells/mm^3^, and his viral load was undetectable a few months ago.

The patient was started on neurosyphilis treatment with benzylpenicillin. Initially, intravenous benzylpenicillin, 400,000 VV every 3 h, was administered. On the second day, as the initial dose was well tolerated, the benzylpenicillin dose was increased to 4 mln every 4 h. Since the patient was immunocompromised and a more severe disease course was anticipated, it was decided to extend the treatment beyond 14 days. On day 19 of high-dose benzylpenicillin therapy, the patient developed a fever of 39 °C. He also complained of abdominal pain, general weakness, and a slight rash and redness at the catheter site. A repeated blood analysis revealed leukopenia (0.7 × 10^9^/L), neutropenia (0.2 × 10^9^/L), thrombocytopenia (44 × 10^9^/L), hemoglobin 129 g/L, CRB 50.1 mg/L, and procalcitonin 0.91 mcg/L. As there was an influenza season in Lithuania and an outbreak of measles at the time, additional tests for influenza (PCR from a nasopharyngeal swab) and measles (PCR from a nasopharyngeal swab and measles virus IgM and IgG in blood) were performed. However, all tests except for measles IgG were negative. Further, blood and urine cultures were taken, but bacterial growth was not detected. A chest X-ray was repeated, and no changes were detected. An abdominal ultrasound was repeated as well, showing a moderately enlarged spleen (13.1 × 6.1 cm). Hematologist’s advice to discontinue benzylpenicillin due to febrile neutropenia was taken into consideration, and the treatment was changed to ceftazidime (2 g every 8 h) in combination with vancomycin (1 g every 12 h), and filgrastim (300 mcg per day) subcutaneously. The benzylpenicillin treatment was discontinued, and the recommended treatment was started. After two days, the patient’s fever receded, the abdominal pain subsided, and the patient’s well-being improved. The treatment was continued for a total of 7 days, while filgrastim was administered for two days only. A repeated blood analysis revealed the positive outcomes of the treatment (WBC 8 × 10^9^/L; neutrophils 6.01 × 10^9^/L; platelets 80 × 10^9^/L; hemoglobin 129 g/L). The patient was discharged home for further HIV treatment and follow-up.

## 3. Discussion

The diagnosis of neurosyphilis was confirmed by CSF abnormalities along with positive specific tests in both blood and cerebrospinal fluid. A normal blood count with CSF pleocytosis is not unusual in neurosyphilis, which often presents with mild lymphocytic CSF changes, similar to viral and some other bacterial infections (e.g., *Listeria*, *Borrelia*, *Mycobacterium*, *Leptospira*, and *Rickettsia*), rather than the marked changes seen in typical bacterial neuroinfections.

Beta-lactam-induced neutropenia has been observed in cases of infective endocarditis, bowel obstruction, cellulitis, gangrenous appendix, pneumonia, hemangioma, septic arthritis, and pleural empyema [1]. Benzylpenicillin-induced neutropenia in neurosyphilis patients is quite uncommon (approx. 2.42%), while severe neutropenia (as in our case) occurs in only 0.35% [2]. The accompanying symptoms were chills and fever, fatigue, cough, sore throat, diarrhea, and erythematous rash. Blood, sputum, and throat swab cultures did not reveal an infectious etiology (e.g., bacterium and fungus) among the febrile patients [2]. Usually, it is associated with prolonged treatment (10- to 14-day duration), though some data suggest that the duration of beta-lactam therapy prior to the start of neutropenia always exceeds 15 days [3,4]. It is presumed that benzylpenicillin-induced neutropenia in neurosyphilis patients could occur earlier due to exceptionally high daily dose [2]. According to EACS guidelines, neurosyphilis in HIV-positive individuals should be treated for at least 14 days, with the duration extendable on an individual basis [5]. The antibiotics from the penicillin group were shown to induce inhibition of granulopoiesis [6]. According to some theories on genetic and epigenetic modification, benzylpenicillin-induced neutropenia is considered as a dose-related immunological reaction; thus, it may predispose the patient to idiosyncratic drug sensitivity [7,8]. Benzylpenicillin-induced thrombocytopenia is much rarer and only several cases have been documented [2]. Our patient developed thrombocytopenia, possibly induced by penicillin, which is consistent with data reported in the literature [2]. Differential diagnosis of benzylpenicillin-induced febrile neutropenia is difficult due to nonspecific symptoms and a great variety of causes of neutropenia, including infection, other medications, nutrition, hematologic malignancies, rheumatologic disorders, autoimmune neutropenia, etc. [9]. In our case, we attempted to differentiate between the most possible causes of neutropenia such as HIV-induced neutropenia, sepsis, flu (as the patient was hospitalized during the flu season), and measles (as there was a measles outbreak in Lithuania at the time).

In patients with HIV, a decreased blood neutrophil count is closely related to a loss of CD4+ T cells and is correlated with the HIV viral load [10]. According to one study, neutropenia could occur at a median CD4 count of 30 cells/mm^3^ [11]. Due to high CD4 levels (more than 500/mm^3^) and a low viral load (<40 HIV copies/mL), HIV-induced neutropenia was excluded in our patient.

Sepsis was another possible cause of neutropenia in our patient. However, low procalcitonin, CRP, no visible signs of infection on the chest X-ray or abdominal ultrasound, and negative blood and urine cultures allowed to exclude sepsis as a cause. On the other hand, we were not able to fully exclude sepsis due to intravenous catheter-related infection, since the patient had a mild rash and redness around the intravenous catheter. Nevertheless, the quick Sequential Organ Failure Assessment (qSOFA) was 0, suggesting that sepsis diagnosis was not likely indeed. Furthermore, in our case, no other organs and/or systems were damaged, which indirectly confirms that sepsis was very unlikely. Intravenous vancomycin treatment was added to the empirical therapy of neutropenia, due to a possible catheter-related source of infection. As mentioned previously, neither the blood culture nor the urine culture showed positive results.

Flu was one more possible diagnosis we had to rule out. The patient did not recall any recent flu vaccinations he could have received. According to the literature, neutropenia in flu patients is common (around 15.3%), but in the majority of cases, it is mild, with the WBC remaining >1.0 × 10^9^/L, and transient [12]. In our case, flu was excluded after receiving a negative nasopharyngeal swab PCR test.

Between 2018 and 2019, there was a measles outbreak in Lithuania with 834 confirmed measles cases [13]. Although measles symptoms include a typical rash, fever, neutropenia, and conjunctivitis, some measles cases without rash and associated with worse outcomes have been reported, mostly in patients with immunosuppression [14]. The patient’s measles vaccination status and his history of previous measles infection were unknown, and so the patient could potentially be infected with measles virus. To test for this possibility, measles IgM, measles IgG, and measles PCR were performed. PCR appeared to be negative and the IgM test was negative. However, the IgG analysis was positive, suggesting that the patient was vaccinated or previously infected with measles virus.

For differential diagnosis, it is important to consider the possibility of a Jarisch–Herxheimer reaction. In our case, anticipating this, we initiated treatment with lower doses of penicillin and increased to high doses as it was well tolerated (Table 1).

Febrile neutropenia therapy with ceftazidime, vancomycin, and G-CSF was prescribed for this high-risk patient (inpatient status at the time of development of fever, possible catheter-related infection, HIV infection) [15]. As there are no clear guidelines for the treatment of neutropenic non-cancer patients, it could be argued whether our decision was the best empirical approach. Since the duration of syphilis treatment was sufficient, retreatment was not considered necessary.

In the cases described in the literature, one patient was treated with antihistamines and methylprednisolone (40 mg daily) instead of benzylpenicillin. His symptoms and CBC normalized by day 8. Three months later, intravenous ceftriaxone (1.0 g every 12 h for 15 days) was reintroduced without complications. Two other patients received only symptomatic and supportive care without G-CSF, glucocorticosteroids, or broad-spectrum antibiotics. Their CBCs normalized within four and five days after stopping benzylpenicillin. Intravenous ceftriaxone, given three months later, did not cause neutropenia. The remaining 11 patients with mild-to-moderate neutropenia completed the 14-day benzylpenicillin course under close CBC monitoring. None experienced serious complications, and neutrophils normalized within seven days. Some received a second benzylpenicillin course three months later, which either did not cause neutropenia or resulted in asymptomatic recurrence [2].

This case report has some limitations. Because these infections are rarely diagnosed in our country, we could not rule out rickettsial co-infection in the case of unexplained eruptive fever. A bone marrow test was not performed, so we cannot fully confirm the cause of neutropenia and thrombocytopenia. Although HIV was well controlled, we cannot rule out that the virus or another hidden infection (like parvovirus B19) may have contributed. However, the blood counts returned to normal soon after stopping penicillin, which supports a drug-related reaction.

## 4. Conclusions

This case highlights that, although rare, high-dose benzylpenicillin-induced neutropenia should be considered in neurosyphilis patients when there is no evidence of other causes for neutropenia. Early detection and proper treatment are needed to prevent this dangerous condition from deteriorating further and to minimize mortality.

## Figures and Tables

**Table 1:** Differential diagnosis of neutropenia.

Suspected Cause	Suspicion	Exclusion
Penicillin-induced neutropenia	Temporal association between benzylpenicillin administration and onset of neutropenia.	Clinical improvement and normalization of CBC after drug withdrawal.
HIV-induced neutropenia	HIV infection is a known cause of neutropenia, especially with low CD4 counts.	Patient had high CD4+ T cell count (>500/mm^3^) and undetectable viral load (<40 copies/mL).
Sepsis (including catheter-related)	Mild rash and redness around IV catheter; sepsis is a known cause of neutropenia.	Normal CRP and procalcitonin, negative blood and urine cultures, no organ dysfunction, qSOFA score = 0, and unremarkable imaging.
Influenza-associated neutropenia	Flu can cause mild transient neutropenia, especially in unvaccinated individuals.	Negative nasopharyngeal swab PCR for influenza virus.
Measles	History of outbreak in region; measles can cause rash, fever, and neutropenia.	Negative measles PCR and IgM; positive IgG suggests past infection or vaccination.
Jarisch–Herxheimer reaction	Known reaction in syphilis treatment with penicillin; may involve transient neutropenia.	Considered early in management—low initial penicillin dose used and gradually increased; no clinical signs consistent with this reaction observed.

## Data Availability

The dataset is available from the authors upon request.

## Abbreviations

The following abbreviations are used in this manuscript:

CRP	C-reactive protein
CSF	Cerebrospinal fluid
PCR	Polymerase chain reaction
WBC	White blood cell
qSOFA	quick Sequential Organ Failure Assessment
